# Assessment of a pretomanid analogue library for African trypanosomiasis: Hit-to-lead studies on 6-substituted 2-nitro-6,7-dihydro-5*H*-imidazo[2,1-*b*][1,3]thiazine 8-oxides

**DOI:** 10.1016/j.bmcl.2017.10.067

**Published:** 2018-01-15

**Authors:** Andrew M. Thompson, Andrew J. Marshall, Louis Maes, Nigel Yarlett, Cyrus J. Bacchi, Eric Gaukel, Stephen A. Wring, Delphine Launay, Stephanie Braillard, Eric Chatelain, Charles E. Mowbray, William A. Denny

**Affiliations:** a Auckland Cancer Society Research Centre, School of Medical Sciences, The University of Auckland, Private Bag 92019, Auckland 1142, New Zealand; b Laboratory for Microbiology, Parasitology and Hygiene, Faculty of Pharmaceutical, Biomedical and Veterinary Sciences, University of Antwerp, Universiteitsplein 1, B-2610 Antwerp, Belgium; c Haskins Laboratories, Pace University, NY 10038, USA; d Scynexis, Inc., Research Triangle Park, NC 27713, USA; e Drugs for Neglected Diseases initiative, 15 Chemin Louis Dunant, 1202 Geneva, Switzerland

**Keywords:** African trypanosomiasis, Pretomanid, Library screening, *In vivo* efficacy, Pharmacokinetics, Nitroimidazole

## Abstract

A 900 compound nitroimidazole-based library derived from our pretomanid backup program with TB Alliance was screened for utility against human African trypanosomiasis (HAT) by the Drugs for Neglected Diseases *initiative*. Potent hits included 2-nitro-6,7-dihydro-5*H*-imidazo[2,1-*b*][1,3]thiazine 8-oxides, which surprisingly displayed good metabolic stability and excellent cell permeability. Following comprehensive mouse pharmacokinetic assessments on four hits and determination of the most active chiral form, a thiazine oxide counterpart of pretomanid (**24**) was identified as the best lead. With once daily oral dosing, this compound delivered complete cures in an acute infection mouse model of HAT and increased survival times in a stage 2 model, implying the need for more prolonged CNS exposure. In preliminary SAR findings, antitrypanosomal activity was reduced by removal of the benzylic methylene but enhanced through a phenylpyridine-based side chain, providing important direction for future studies.

## Graphical abstract

**Figure:**
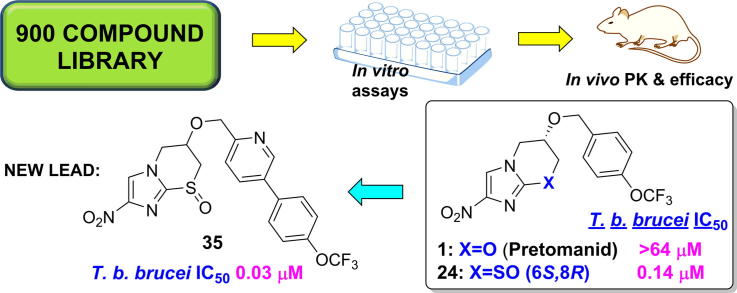

Human African trypanosomiasis (HAT, also known as sleeping sickness) is a particularly lethal neglected tropical disease that is endemic in remote sub-Saharan Africa.^1^ HAT arises from infection by two subspecies of the kinetoplastid parasite *Trypanosoma brucei* (*T. b. gambiense* and *T. b. rhodesiense*), which are transmitted through the bite of tsetse flies.^2^ Because symptoms of the initial bloodstream stage are fairly mild and non-specific (e.g., headache, fever, weakness), the disease often progresses to the potentially fatal CNS stage characterised by neurological and psychiatric disorders before treatment is sought.^1^, ^3^ However, there are pitifully few available drugs for late stage HAT, and all require hospitalization.^3^, ^4^ The antiquated first-line remedy melarsoprol (**1**, see Fig. 1) is highly toxic, causing death in ∼5% of patients, and is increasingly less effective due to drug resistance.^4^, ^5^ Eflornithine (**2**) is less toxic but more costly and cumbersome to administer and is ineffective against *T. b. rhodesiense* (<5% of total cases).^6^ Combination of **2** with nifurtimox (**3**) (NECT) has recently led to reduced cost and workload without compromising efficacy,^7^ but similar issues (adverse effects and parenteral administration) plus a lack of CNS penetration limit the two early stage drugs, pentamidine and suramin.^3^, ^4^, ^5^ Thus, there is a compelling need for more universally effective, safe and affordable oral therapies. Two promising new agents are now in phase II/III clinical trials; fexinidazole (**4**) and oxaborole SCYX-7158 (**5**).^8^, ^9^ Nevertheless, in order to mitigate development risks and minimise the emergence of drug resistance, it remains essential to develop a pipeline of novel agents with unique mechanisms of action.^4^

**Fig. 1:**
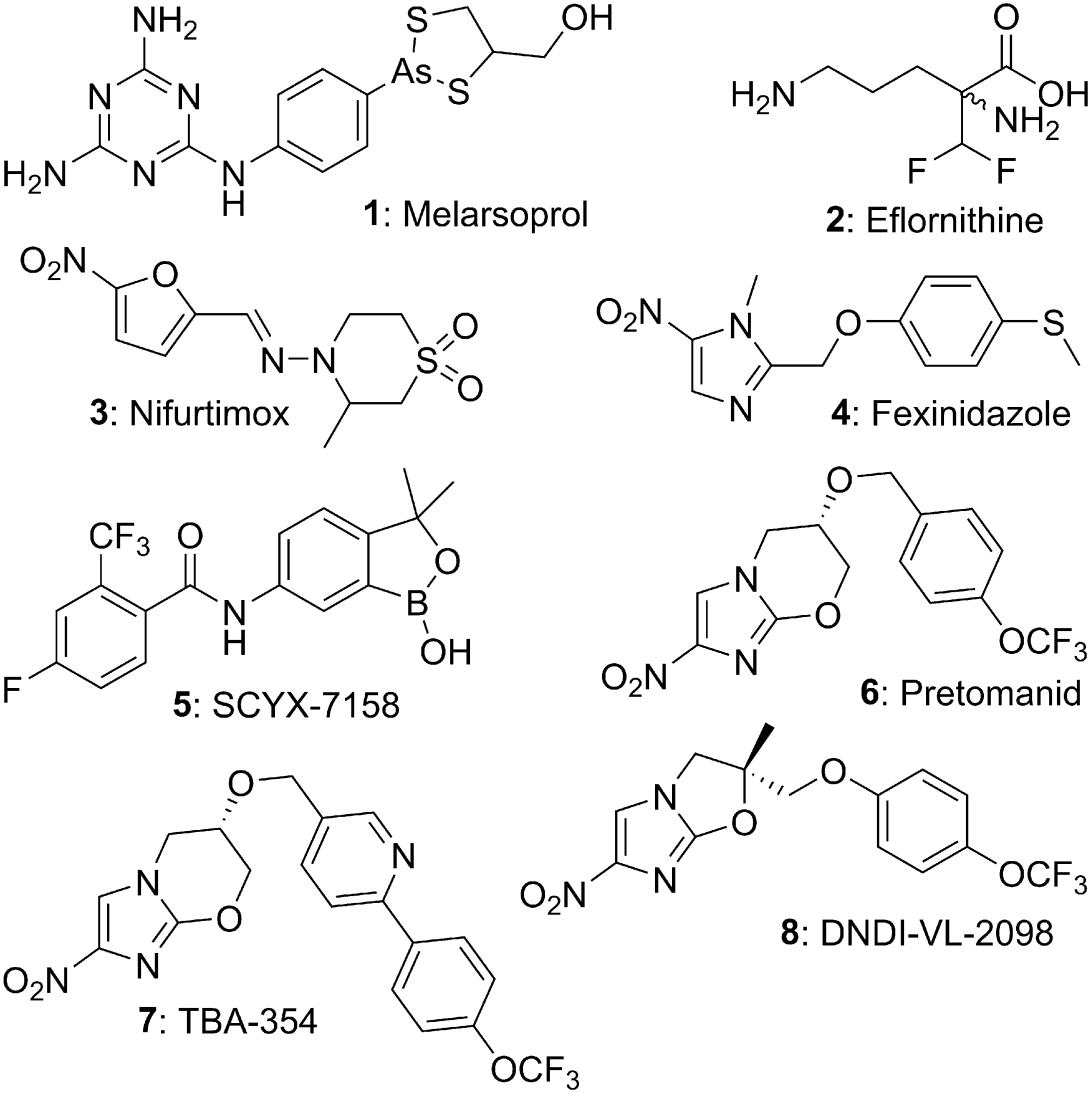
Various antitrypanosomal, antitubercular, or antileishmanial agents.

The nitroimidazooxazine pretomanid (PA-824, **6**) has demonstrated excellent bactericidal efficacy in phase II clinical studies for tuberculosis (TB), stimulating its further appraisal in new drug combination trials.^10^ Within a comprehensive backup program, in collaboration with the TB Alliance, we generated a library of more than 1000 compounds, whose assessment led to the advancement of a second generation TB candidate (TBA-354, **7**) into phase I clinical evaluation.^11^ We recently disclosed that phenotypic screening of some early examples against kinetoplastid diseases by the Drugs for Neglected Diseases *initiative* (DND*i*) unexpectedly enabled the discovery of DNDI-VL-2098 (**8**) as a preclinical lead for visceral leishmaniasis.^12^ Unfortunately, **8** exhibited poor potency against *T. b. brucei* (IC_50_ 53 µM) and it was reported^13^ that **6** also had weak activity versus this parasite (IC_50_ 38 µM). However, unlike fexinidazole (**4**), **6** did not display cross-resistance to nifurtimox (**3**),^13^ indicating that it is not activated by the same type I nitroreductase employed by **3** and **4** (implying a different mechanism of action). Therefore, as part of a wider search for improved development candidates for HAT, ∼900 analogues of **6** were screened by DND*i* and several promising hits were unearthed. Herein, we reveal initial *in vitro*/*in vivo* profiling data on these hits, and findings from a preliminary SAR study of a nitroimidazothiazine oxide lead.

Medium-throughput screening and follow-up IC_50_ testing at Scynexis^14^ identified 48 active hits (IC_50_ < 3 µg/mL), of which 19 were initially considered to be of potential interest (mean IC_50_ < 1 µg/mL, with selectivity index > 10). Intriguingly, the most active compounds (**9**–**12**; Table 1) were either 2-nitroimidazothiazine oxides^15^ or 6-nitroimidazothiazole oxides,^16^ but a wide variety of other structures, including extended side chain analogues of **6**, featured in this set. Since good CNS penetration is a critical requirement for the effective treatment of stage 2 HAT,^9^ the 10 most potent hits were first evaluated for cell permeability in the MDCK-MDR1 assay. In this test system, apparent permeability (P_app_) values ≥150 nm/s are indicative of high brain penetration potential provided that the transport is not affected by P-gp inhibition^17^ (necessitating an absorption quotient in the range −0.1 to 0.1). Unsurprisingly, the compound with a triaryl side chain (**13**, MW > 500) lacked any significant permeability (P_app_ < 0.8 nm/s), while four others (**10**, **14**, **16**, and **18**)^15^, ^18^, ^19^ gave only modest permeability values (P_app_ < 150 nm/s) and were suggested to be P-gp substrates (absolute AQs ≥ 0.3).^20^ On the basis of results from this training set, additional hits were selected for assessment (**19**–**23**)^15^, ^16^, ^19^ and, pleasingly, all of these demonstrated a high propensity to cross the blood-brain barrier.

**Table 1:** Inhibitory potency, metabolic stability, aqueous solubility, and MDCK-MDR1 cell permeability for 15 screening hits against *T. b. brucei.*

**Figure:**
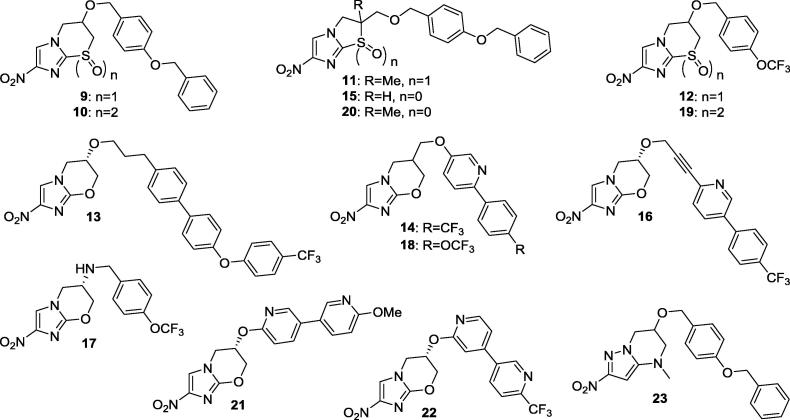

Compd	IC_50_ (µg/mL)^a^		Selectivity Index	Mouse S9^b^	Solubility^c^	Permeability (nm/s)^d^		
	*T. b. brucei*	L929		t_1/2_ (min)	(µg/mL)	P_app_	P_app_ + 918	AQ
**9**	0.015 ± 0.005	>10	>667	173	5.4	771	799	0.035
**10**	0.033 ± 0.018	>10	>303	ND	ND	36.9	254	0.85
**11**	0.13 ± 0.04	>10	>77	50	1.3	651	637	−0.022
**12**	0.16 ± 0.07	>10	>63	>350	39	798	848	0.059
**13**	0.23 ± 0.04	>10	>43	ND	ND	<0.8	<0.8	ND
**14**	0.28 ± 0.11	>10	>36	ND	ND	117	71	−0.64
**15**	0.46 ± 0.21	>10	>22	67	1.2	583	574	−0.016
**16**	0.53 ± 0.23	>10	>19	ND	ND	35	49	0.29
**17**	0.78 ± 0.01	>10	>13	>350	>72	804	789	−0.019
**18**	0.83 ± 0.41	>10	>12	ND	ND	74	37	−1
**19**	0.90 ± 0.40	>10	>11	298	10	624	656	0.049
**20**	2.2 ± 0.7	>10	>4.5	31	1.2	465	465	0
**21**	2.7 ± 0.5	>10	>3.7	239	18	769	767	−0.003
**22**	2.8 ± 0.2	>10	>3.6	146	10	793	758	−0.046
**23**	3.0 ± 0.9	>10	>3.3	38	2.4	363	332	−0.093

a IC_50_ values for inhibition of the growth of *T. b. brucei* 427 or for cytotoxicity toward L929 mouse fibroblasts. Each value is the mean of ≥2 independent determinations ± standard deviation.

b Half-life in mouse liver S9 fraction (ND: not determined).

c Kinetic aqueous solubility in pH 7.4 PBS.

d Permeability of compounds (at 3 µM) in an MDCK-MDR1 cell monolayer assay (A to B) in the presence or absence of the P-gp inhibitor GF120918 (2 µM); AQ is the absorption quotient, as defined by the equation: AQ = (P_app_ + 918 − P_app_)/P_app_ + 918. In this assay, the CNS positive drug propranolol gave P_app_ 556 nm/s and **4** had P_app_ 732 nm/s.

In order to determine the suitability of the more permeable hits for *in vivo* efficacy studies, we first measured their aqueous solubilities, and their tendencies to metabolise, following a 1 h incubation with CD-1 mouse liver S9 subcellular fractions.^14^ Here, the most poorly soluble compounds (**11**, **15**, **20**, and **23**) were also found to be the least stable, displaying half-lives of less than 70 min. Overall, the 2-nitroimidazothiazine oxides **9**, **12** and **19**,^15^ together with the 6-amino-linked analogue of **6** (**17**),^21^ provided the best balance of potency, stability, aqueous solubility and CNS penetration potential. This led us to probe their *in vivo* pharmacokinetic (PK) profiles in mice, examining concentration levels in plasma, whole blood and brain tissue following both intravenous and oral administration (Table 2; for further experimental details, see the Supporting Information).

**Table 2:** Mouse pharmacokinetic parameters for selected compounds.

Compd	Intravenous (0.5–3 mg/kg)^a^				Oral (50–80 mg/kg)^a^				
	CL (L/h/kg)	Vdss (L/kg)	t_1/2_ (h)	AUC_last_ (µg⋅h/mL)	C_max_ (µg/mL)	T_max_ (h)	t_1/2_ (h)	AUC_last_ (µg⋅h/mL)	*F*^b^ (%)
*Plasma*									
**9**	0.97	0.59	0.43	0.505	0.20	2	3.7	0.869	1.4
**12**	0.52	1.6	2.5	2.09	9.3	4	6.9	51.9	55
**17**	6.8	4.7	0.48	0.418	12	1	1.2	20.5	100
**19**	1.0	3.6	2.5	1.80	2.3	2	2.7	18.7	42

*Whole blood*									
**9**	0.87	0.33	0.42	0.489	0.11	2	5.3	0.494	0.8
**12**	0.39	1.7	9.3	2.70	8.6	4	5.8	63.5	52
**17**	2.4	8.1	9.3	1.10	26	0.5	1.5	42.2	100
**19**	0.69	2.8	5.9	2.60	3.0	2	3.3	23.7	37

*Brain*									
**9**	1.5	1.2	1.6	0.304	0.06	2	3.2	0.290	0.8
**12**	0.40	1.3	2.3	2.85	14	4	6.9	71.2	55
**17**	2.9	2.3	0.26	0.962	35	0.5	1.3	50.9	100
**19**	0.51	9.1	14	1.77	2.4	4	3.0	18.8	43

a The corrected intravenous doses for **9**, **12**, **17** and **19** were 0.5, 1.1, 2.9 and 2.0 mg/kg, respectively, and the corresponding oral doses were 62, 50, 78 and 49 mg/kg, respectively.

b Oral bioavailability, determined using dose normalised AUC_last_ values.

The most potent hit (**9**) exhibited an unacceptable PK profile, giving inadequate oral exposure and poor oral bioavailability (<1.5%), consistent with both its low solubility (causing unsatisfactory absorption) and more rapid metabolism. This was unsurprising, as the 4-benzyloxybenzyl analogue of **6** was known to be markedly inferior to **6** against *Mycobacterium tuberculosis in vivo*, despite being an order of magnitude more potent than **6** *in vitro*, due to similar PK issues.^22^ In contrast, the 4-trifluoromethoxybenzyl congener of **9** (**12**) demonstrated the slowest rate of clearance of the four, and a prolonged, high exposure level above the MIC following oral dosing (Fig. 2), with good oral bioavailability (52–55%) at all three sampling sites. Moreover, the high brain:plasma concentration ratio (∼3:2) presented by **12** was encouraging for CNS uptake, as required in the treatment of stage 2 HAT.^14^ The sulfone derivative of **12** (**19**), which was produced to a significant extent in PK samples from the analysis of **12**, showed reduced oral exposure, in accordance with its inferior solubility and faster rate of clearance. Given its weaker potency (5.6-fold vs **12**), these results for **19** were not predictive of good *in vivo* activity, thus *in situ* oxidation of **12** should have a minimal contribution to efficacy. Finally, the most soluble hit **17** (the 6-amino analogue of **6**) was notable for having the best oral bioavailability, with excellent concentration levels observed in brain tissue (2- to 3-fold higher than in plasma). However, this compound also suffered from a high rate of clearance and a rather short oral half-life (1.2–1.5 h), leading to inadequate exposure above the MIC beyond ∼2 h. These latter results mirrored findings from a recently reported PK-PD study of analogues of **6** against TB, in which **17** displayed a 1.3 h oral half-life in mouse lung tissue (in comparison to 4.8 h for **6**),^23^ effectively precluding useful *in vivo* activity. Hence, of the four most promising hits, only the 2-nitroimidazothiazine oxide **12** proved to be suitable for efficacy assessment in the acute infection mouse model of HAT.

**Fig. 2:**
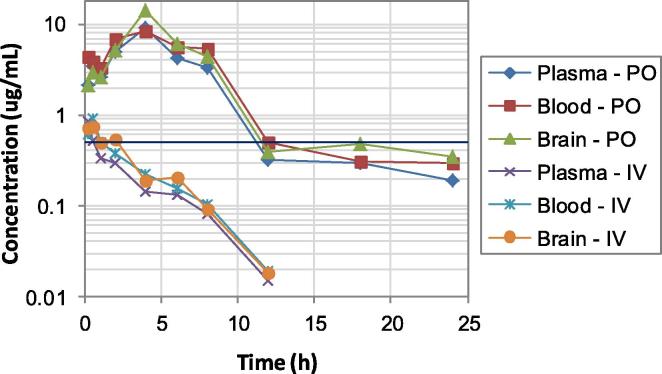
Time vs concentration curves for **12**, following administration to male CD-1 mice (at 50 mg/kg po and 1.1 mg/kg iv). The horizontal line represents the MIC for complete inhibition of visible parasite growth *in vitro*.

One remaining matter to resolve with racemic hit **12** was which one of the four possible stereoisomers was the most active chiral form. This issue was partially clarified through a better optimised resynthesis of **12** (Scheme 1). Following side chain attachment to the racemic alcohol **42**^15^ (93% yield), careful oxidation of thiazine **30** with fresh *m*-CPBA (1.01 equiv) led to a separable mixture of **12** (75%) and a previously unidentified more polar racemic diastereomer **38** (20%) (for experimental details, see the Supporting Information).

**Scheme 1:**
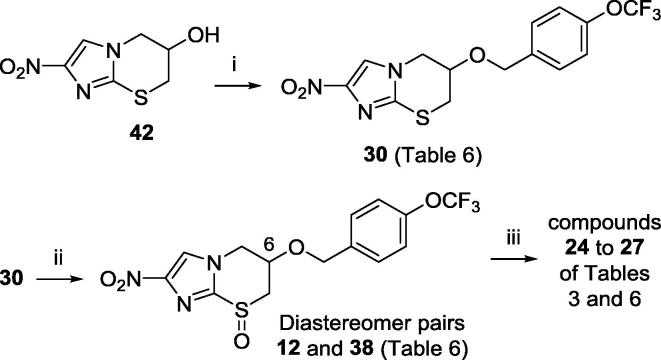
Reagents and conditions: (i) 4-OCF_3_BnBr, NaH, DMF, 20 °C, 160 min (93%); (ii) *m*-CPBA, Na_2_HPO_4_, CH_2_Cl_2_, −10 to 20 °C, 19 h (**12**: 75%, **38**: 20%); (iii) preparative chiral SFC (see text).

The ^1^H NMR spectra of **12** and **38** showed pronounced chemical shift differences for the H-6 resonance in particular, which was ∼0.4 ppm further downfield in the spectrum of **12**. The sulfoxide oxygen in six-membered rings is known to exhibit an axial preference, such that the deshielding effect of the sulfoxide group on axial β-hydrogen atoms has been used to assign relative stereochemistry.^24^ Hence, **12** is postulated to have the sulfoxide oxygen and H-6 in a pseudo-diaxial orientation, placing the (4-OCF_3_)benzyloxy side chain at C-6 in a 1,3-trans relationship to the sulfoxide oxygen. This assignment is supported by the diastereomer ratio (3.5:1) in favour of **12**, which might be rationalised by an expected preference for the C-6 side chain to adopt a pseudoaxial conformation in the thiazine precursor **30** (based on the crystal structure of **6**),^25^ providing a steric disincentive to formation of the cis sulfoxide **38**.

Preparative chiral SFC separation of the enantiomers of **12** (**24** and **26**) and **38** (**25** and **27**) facilitated the assessment of all four stereoisomers (Table 3). The C-6 configuration of **24** and **25** was later firmly established via a known^26^ chiral synthesis. The most active (6*S*) form **24** (IC_50_ 0.07 µg/mL) was 40-fold more potent than cis isomer **25**, and more than 70-fold more potent than its (6*R*) enantiomer **26**. This level of potency compared well with data reported for **5** (IC_50_ 0.29 µg/mL vs *T. b. brucei* 427) in the same Scynexis assay.^9^ Compound **24** also displayed an improved selectivity index (>143), good aqueous solubility (106 µg/mL), and excellent metabolic stability following a 1 h exposure to human and mouse liver microsomes (respectively, 82% and 96% parent remaining).

**Table 3:** *In vitro* potency and microsomal stability of the enantiomers of sulfoxides **12** and **38** (by convention,^30^ the sulfur-oxygen double bond has been depicted as a chiral single bond).

**Figure:**
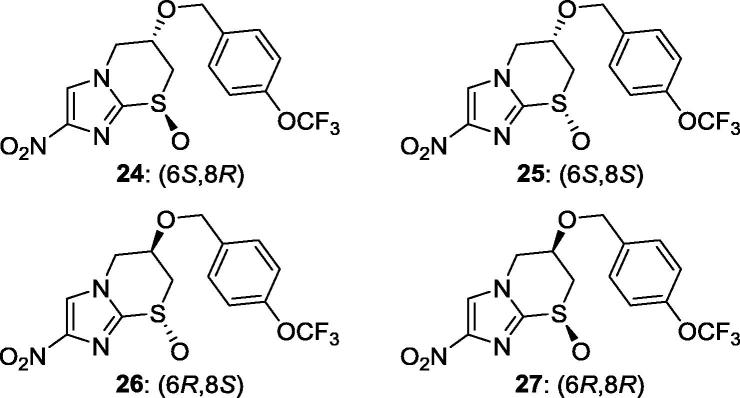

Compd	IC_50_ (µg/mL)^a^		Microsomes^b^ (% remaining at 1 h)	
	*T. b. brucei*	L929	Human	Mouse
**24**	0.070 ± 0.005	>10	82	96
**25**	2.8 ± 0.4	>10	93	93
**26**	>5	>10	91	26
**27**	>5	>10	88	89

a IC_50_ values for inhibition of the growth of *T. b. brucei* 427 or for cytotoxicity toward L929 mouse fibroblasts. Each value is the mean of 2 independent determinations ± standard deviation.

b Pooled human or CD-1 mouse liver microsomes.

Therefore, **24** was examined in a stage 1 HAT mouse model.^14^ Briefly, dosing was orally once daily for four days, starting 24 h postinfection, and parasitemia was assessed weekly via tail vein blood smears (see the Supporting Information). Excellent activity was observed (Table 4), with **24** providing complete cures (i.e. parasite free blood smears after >30 days) to all mice at doses as low as 5 mg/kg, similar to the control drug pentamidine (given i.p. at 2 mg/kg), whereas the vehicle only mice died on day 7. The efficacy seen with **24** in this model was equivalent to the level of activity reported for **5** and ∼20-fold superior to the results described for fexinidazole (**4**),^8^, ^9^ stimulating further evaluation of this lead in a stage 2 HAT mouse model.^14^ Here, oral dosing of **24** (at 12.5 to 50 mg/kg *once daily* for seven days from day 21 postinfection) led to significant increases in survival times in comparison to untreated controls (66–70 days vs 31 days; Table 5), although cure rates were inadequate (0–20%). In contrast, **5** was 100% curative in the same CNS model at a dosage of 25 mg/kg once daily for 7 days,^9^ while **4** gave an 88% cure rate in a comparable model when administered orally at 200 mg/kg once daily for 5 days.^8^

**Table 4:** *In vivo* activity of **24** in a *T. b. brucei* (EATRO 110) acute infection mouse model.

Compd	Dosage^a^ (mg/kg)	Mean survival (days)	Cured/Total	Cured (%)
**24**	50	>30	5/5	100
**24**	25	>30	5/5	100
**24**	12.5	>30	5/5	100
**24**	5	>30	4/4	100
**24**	2.5	13	1/4	25
**24**	1.25	7.5	0/4	0
Pentamidine	2	>30	3/3	100
Vehicle^b^		7	0/3	0

a Dosing of **24** was orally, once daily for 4 days consecutively, while pentamidine was dosed i.p. once daily for the same period.

b Vehicle for **24**: 0.8% CMC, 0.1% SDS in water.

**Table 5:** *In vivo* activity of **24** in a *T. b. brucei* (TREU 667) CNS infection mouse model.

Compd	Dosage^a^ (mg/kg)	Mean relapse time (days)	Cured/Total	Cured (%)
**24**	50	66	0/10	0
**24**	25	70	2/10	20
**24**	12.5	45	0/8	0
Berenil	10 (D4)^b^		5/5	100
Berenil	10 (D21)^b^	41	0/5	0
Vehicle^c^		31	0/5	0

a Dosing of **24** was orally, once daily for 7 d consecutively, starting on day 21 postinfection.

b Single i.p. dose on day 4 or day 21.

c Vehicle for **24**: 0.8% CMC, 0.1% SDS in water.

Detailed *in vivo* studies in the benzoxaborole 6-carboxamide class have revealed that efficacy in the CNS model is heavily dependent upon the maintenance of drug concentrations in the brain for at least 15 h at levels above the MIC (defined as the lowest compound concentration that completely inhibits visible parasite growth *in vitro* after a 72 h incubation).^27^, ^28^ Thus, a more potent analogue of **5** without the *gem*-dimethyl group (SCYX-6759) required an oral dosing regimen of 50 mg/kg *twice* daily (b.i.d.) in order to obtain an 83% cure rate of the CNS infection,^14^ due to the shorter time that its brain concentration level was at or above the MIC (∼12 h vs ∼ 24 h for **5** at 25 mg/kg^9^). These findings imply that a similar oral dosing regimen of 50 mg/kg b.i.d. might be required to achieve useful efficacy for **24** in the stage 2 HAT model (via more prolonged CNS exposure). Nevertheless, these initial *in vivo* results with **24** were still regarded as encouraging, and indicated that 2-nitro-6,7-dihydro-5*H*-imidazo[2,1-*b*][1,3]thiazine 8-oxides merited further investigation as potential treatments for HAT. Specifically, as illustrated with benzoxaboroles, we considered the possibility of designing new analogues of **24** having improved potency and extended CNS exposure. On the basis of the results above and insights from previous SAR studies directed at developing a backup TB candidate to the structurally related nitroimidazooxazine **6**,^19^, ^29^ we devised two preliminary strategies for optimisation of the side chain of **24**: a) removal of the benzylic methylene group and b) insertion of a proximal pyridine ring (*cf*. **7**). Notably, both strategies had the potential to improve metabolic stability,^19^, ^29^ leading to longer *in vivo* half-lives and better exposure levels. Furthermore, to mitigate any reduction in solubility with the first approach, we also proposed the preparation of a trifluoromethylpyridinyl ether analogue (*cf*. **21** and **22**).

The synthetic methods employed to prepare the new nitroimidazothiazine derivatives (**28**, **29**, **32**–**37**, **39**–**41**) are outlined in Scheme 2. Mitsunobu coupling of the orthogonally diprotected triol **44**^31^ with 4-(trifluoromethoxy)phenol and conversion of the product **45** to iodide **47** (via successive hydrogenolysis of the benzyl ether and iodination using I_2_/PPh_3_/imidazole) set the stage for the preparation of phenyl ether **28** (Scheme2A). Thus, base-assisted alkylation of 2-chloro-4-nitroimidazole with iodide **47**, followed by desilylation (TBAF), provided the key alcohol **49** (73%, 2 steps). Then, reaction of the tosylate derivative of **49** (**50**) with the lithium salt of triisopropylsilanethiol and treatment of the crude product with TBAF enabled cyclisation to thiazine **28** (31%). Finally, careful oxidation of **28** with fresh *m*-CPBA (1.2 equiv) led to a separable mixture of sulfone **40** (11%) and the diastereomeric sulfoxides **33** and **36** (82% and 2%), where the sizeable diastereomer ratio (dr ∼ 34:1) was in accordance with the greater steric hindrance induced by this phenoxy side chain. Thiazine pyridinyl ether **29** was more directly accessed via a sodium hydride-induced S_N_Ar reaction of thiazine alcohol **42**^15^ with 2-chloro-5-trifluoromethylpyridine (**52**) (69%; Scheme2B), while alternative alkylation of **42** with 5-bromo-2-(bromomethyl)pyridine^29^ (**53**), followed by Suzuki coupling with 4-(trifluoromethoxy)phenylboronic acid, furnished the extended side chain thiazine **32** (35% over 2 steps; Scheme2C). However, whereas *m*-CPBA oxidation of **29** proved straightforward, similar oxidation of **32** was complicated by the formation of smaller amounts of pyridine *N-*oxide derivatives, such that only the sulfoxides **35** and **39** (55% and 8%) could be obtained. All new compounds (Table 6) were characterised by ^1^H NMR, MS, melting point, and combustion analysis (or HRMS and HPLC); full synthetic procedures and characterisation data have been provided in the Supporting Information.

**Scheme 2:**
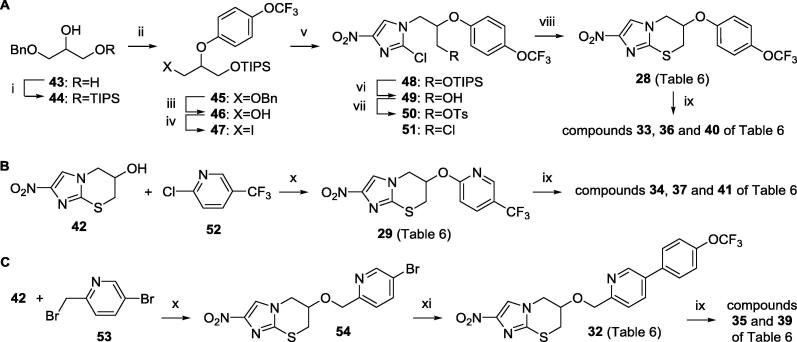
Reagents and conditions: (i) TIPSCl, imidazole, DMF, 20 °C, 3 d (92%); (ii) 4-OCF_3_PhOH, PPh_3_, DEAD, THF, 0–20 °C, 4.5 d (75%); (iii) H_2_, 10% Pd-C, EtOH, EtOAc, 2 d (98%); (iv) I_2_, PPh_3_, imidazole, CH_2_Cl_2_, 20 °C, 15 h (98%); (v) 2-chloro-4-nitroimidazole, K_2_CO_3_, DMF, 85 °C, 64 h (88%); (vi) TBAF, THF, 0–5 °C, 5 h (83%); (vii) TsCl, pyridine, 0–20 °C, 25 h (**50**: 84%; **51**: 9%); (viii) LiSTIPS, THF, −78 to 20 °C, 2 d, then TBAF, THF, 20 °C, 13 h (31%); (ix) *m*-CPBA, Na_2_HPO_4_, CH_2_Cl_2_, −10 to 20 °C, 23–52 h (**33**: 82%, **36**: 2%, **40**: 11%; **34**: 60%, **37**: 11%, **41**: 28%; **35**: 55%, **39**: 8%); (x) NaH, DMF, 0–20 °C, 3–3.5 h (**29**: 69%; **54**: 79%); (xi) 4-OCF_3_PhB(OH)_2_, toluene, EtOH, DMF, 2M Na_2_CO_3_, Pd(dppf)Cl_2_ under N_2_, 84 °C, 4.5 h (44%).

**Table 6:** *In vitro* antiparasitic activities and calculated lipophilicities of 2-nitro-6,7-dihydro-5*H*-imidazo[2,1-*b*][1,3]thiazine analogues.

**Figure:**
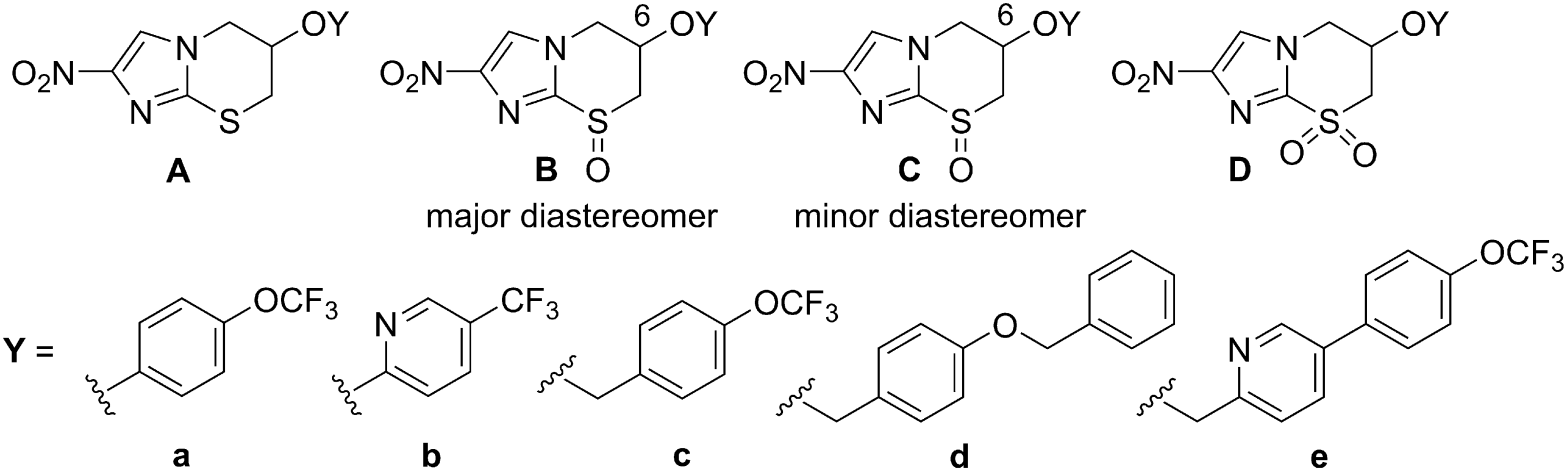

Compd	Form	CLogP^a^	IC_50_ (µM)^b^				
			*T. b. bruc*	*T. b. rhod*	*T. cruzi*	*L. inf*	MRC-5
**28**	Aa	2.83	40	59	2.2	7.0	51
**29**	Ab	2.68	>64	>64	5.3	10	>64
**30**^c^	Ac	3.05	44	36	1.1	55	>64
**31**^c^	Ad	3.67	1.4	1.2	0.49	45	>64
**32**	Ae	3.39	4.3	2.1	1.4	7.5	21
**33**	Ba	1.19	1.6	0.98	2.1	7.0	23
**34**	Bb	1.05	1.2	0.51	4.1	13	>64
**12**^c^	Bc	1.42	0.27	0.25	1.5	16	60
**24**^d^	Bc^e^	1.42	0.14	0.13	1.4	10	50
**26**	Bc^f^	1.42	34	7.3	6.5	41	>64
**9**^c^	Bd	2.04	0.030	0.027	0.12	3.4	64
**35**	Be	1.76	0.030	0.023	0.067	0.41	16
**36**	Ca	1.19	55	27	7.7	>64	>64
**37**	Cb	1.05	17	9.9	12	41	30
**38**	Cc	1.42	5.6	5.1	9.1	>64	>64
**25**	Cc^e^	1.42	5.6	1.9	13	>64	>64
**27**	Cc^f^	1.42	16	4.3	13	48	>64
**39**	Ce	1.76	0.075	0.10	0.14	2.0	54
**40**	Da	1.50	15	13	3.5	32	>64
**41**	Db	1.36	19	11	2.6	30	46
**19**^c^	Dc	1.73	1.1	0.94	1.6	>64	>64
**10**^c^	Dd	2.35	0.097	0.027	0.35	7.3	>64

a Calculated lipophilicities derived from ACD LogP software (v 14.04).

b IC_50_ values for inhibition of growth of the parasites *T. b. brucei* 427, *T. b. rhodesiense*, *Trypanosoma cruzi*, and *Leishmania infantum*, or for cytotoxicity toward human lung fibroblasts (MRC-5 cells). Each value is the mean of 2 to 5 independent determinations. For complete results (mean ± SD), refer to the Supporting Information.

c Ref. 15.

d Ref. 26.

e (6*S*)-Enantiomer.

f (6*R*)-Enantiomer.

The new compounds and relevant comparators were screened at the University of Antwerp against a panel of four protozoan parasites (*T. b. brucei*, *T. b. rhodesiense, T. cruzi*, and *L. infantum*); cytotoxic effects on human lung fibroblasts (MRC-5 cells, the host for *T. cruzi*) were also assessed.^32^ In all cases, recorded data (Table 6) are mean values derived from two or more independent experiments. For the parent thiazines (**28**–**32**), antitrypanosomal potency was enhanced by an order of magnitude with biaryl side chains (d and e), and this SAR pattern was maintained for the considerably less lipophilic major sulfoxide disastereoisomers (Ba-e), where **35** was the most impressive new HAT lead (*T. b. brucei* IC_50_ 0.030 µM). This lead was also highly effective against Chagas disease (*T. cruzi* IC_50_ 0.067 µM) and was the only compound to display submicromolar antileishmanial activity (*L. inf* IC_50_ 0.41 µM). In contrast, shorter linked aryl ether sulfoxides **33** and **34** were 4- to 6-fold less potent than the initial hit **12** against *T. b. brucei*, while their sulfone derivatives (**40** and **41**) were an order of magnitude inferior to sulfone **19**, indicating that the original (OCH_2_) linkage was best. In comparison to **12** (the racemic form of lead **24**), racemic sulfoxide **35** displayed a 9-fold greater potency against *T. b. brucei*, an 11-fold higher potency against *T. b. rhodesiense*, and a 2.4-fold better selectivity index (MRC-5 IC_50_ > 500 times larger than the HAT IC_50_). Compound **35** also demonstrated acceptable aqueous solubility (9.9 µg/mL at pH 7 and 1260 µg/mL at pH 1), high permeability potential without P-gp mediated efflux (MDCK-MDR1 cell P_app_ A-B/B-A 117/182 nm/s *cf*. P_app_ A-B of 197 nm/s for the CNS positive drug propranolol in the same assay) and very good stability toward human and mouse liver microsomes (respectively, 78% and 72% parent remaining after 1 h). While we have not yet had the opportunity to evaluate **35** beyond this stage, these promising results certainly point to the viability of this SAR approach to provide useful new HAT leads.

In summary, this investigation set out to evaluate a nitroimidazole-based compound library related to pretomanid for possible utility against HAT. Although the hit rate was low (∼2%), several compounds displayed good metabolic stability, adequate solubility, and excellent CNS penetration potential. Comprehensive mouse pharmacokinetic studies of three oxidised nitroimidazothiazines and a 6-amino-linked analogue of pretomanid identified the racemic thiazine oxide **12** as a suitable candidate for *in vivo* efficacy studies. The most potent stereoisomer of **12** (**24**) was indeed highly efficacious in the stage 1 HAT mouse model with once daily oral dosing (similar to oxaborole **5**), but was less effective in a stage 2 model. While it seemed reasonable to speculate that more frequent dosing with **24** should achieve better outcomes in this latter model, we also envisaged the generation of new analogues with higher potency and longer half-lives. In preliminary SAR work, we noted that removal of the benzylic methylene was disfavoured but that adding a proximal pyridine ring (**35**) enhanced potency while broadly retaining other essential properties. These additional findings are very encouraging and provide a rational foundation for further development of this interesting class of antitrypanosomal agents.

## A Supplementary data

Supplementary data Experimental procedure and spectral data.

### MOL files

The following ZIP file contains the MOL files of the most important compounds referred to in this article.

MOL files ZIP file containing the MOL files of the most important compounds in this article.
