# Supplementary material for: Assessment of a pretomanid analogue library for African trypanosomiasis: Hit-to-lead studies on 6-substituted 2-nitro-6,7-dihydro-5H-imidazo[2,1-b][1,3]thiazine 8-oxides
Source: Bioorg Med Chem Lett. 2018 Jan 15;28(2):207–13. doi: 10.1016/j.bmcl.2017.10.067 (PMC5840523; doi:10.1016/j.bmcl.2017.10.067)
Supplement: Supplementary data — Experimental procedure and spectral data. [file mmc1.pdf]

Supporting Information

Assessment of a pretomanid analogue library for African trypanosomiasis: Hit-to-lead studies on 6-substituted 2-nitro-6,7-dihydro-5*H*-imidazo[2,1-*b*][1,3]thiazine 8-oxides

Andrew M. Thompson, Andrew J. Marshall, Louis Maes, Nigel Yarlett, Cyrus J. Bacchi, Eric Gaukel, Stephen A. Wring, Delphine Launay, Stephanie Braillard, Eric Chatelain, Charles E. Mowbray, and William A. Denny

Contents:

Table S1. Complete *in vitro* antiparasitic data for the compounds of Table 6 (p S2)

Experimental procedures and characterizations for the compounds of Table 6 (pp S3-S10)

Protocols for *in vitro* antiparasitic and cytotoxicity testing, microsomal and S9 stability, permeability, solubility, mouse pharmacokinetics, and *in vivo* efficacy assays (pp S10-S11)

References for Supporting Information (p S12)

Table S2. Combustion analysis data for compounds of Table 6 and intermediates (p S13)

Table S1. Complete *in vitro* antiparasitic data for the compounds of Table 6

	IC_{50}[a,b] (μM)				
compd	*T. b. bruc*	*T. b. rhod*	*T. cruzi*	*L. inf*	MRC-5
**28**	40 ± 14	59 ± 4	2.2 ± 0.4	7.0 ± 1.2	51 ± 17
**29**	>64	>64	5.3 ± 1.0	10 ± 2	>64
**30**	44 ± 3	36 ± 4	1.1 ± 0.2	55 ± 10	>64
**31**	1.4 ± 0.6	1.2 ± 0.5	0.49 ± 0.31	45 ± 27	>64
**32**	4.3 ± 2.3	2.1 ± 0.5	1.4 ± 0.4	7.5 ± 3.7	21 ± 9
**33**	1.6 ± 0.1	0.98 ± 0.49	2.1 ± 0.1	7.0 ± 1.1	23 ± 8
**34**	1.2 ± 0.5	0.51 ± 0.16	4.1 ± 0.6	13 ± 5	>64
**12**	0.27 ± 0.10	0.25 ± 0.10	1.5 ± 0.7	16 ± 3	60 ± 4
**24**	0.14 ± 0.02	0.13 ± 0	1.4 ± 0.6	10 ± 3	50 ± 8
**26**	34 ± 11	7.3 ± 3.4	6.5 ± 0.4	41 ± 5	>64
**9**	0.030 ± 0	0.027 ± 0.005	0.12 ± 0.01	3.4 ± 1.5	64 ± 1
**35**	0.030 ± 0	0.023 ± 0.009	0.067 ± 0.005	0.41 ± 0.19	16 ± 2
**36**	55 ± 10	27 ± 18	7.7 ± 0.9	>64	>64
**37**	17 ± 2	9.9 ± 2.6	12 ± 0	41 ± 6	30 ± 4
**38**	5.6 ± 0.1	5.1 ± 0.3	9.1 ± 2.3	>64	>64
**25**	5.6 ± 0.3	1.9 ± 0.3	13 ± 2	>64	>64
**27**	16 ± 4	4.3 ± 0.1	13 ± 2	48 ± 5	>64
**39**	0.075 ± 0.045	0.10 ± 0.03	0.14 ± 0.09	2.0 ± 0.4	54 ± 11
**40**	15 ± 5	13 ± 6	3.5 ± 1.3	32 ± 0	>64
**41**	19 ± 10	11 ± 3	2.6 ± 0.6	30 ± 4	46 ± 5
**19**	1.1 ± 0.4	0.94 ± 0.28	1.6 ± 0.7	>64	>64
**10**	0.097 ± 0.047	0.027 ± 0.005	0.35 ± 0.18	7.3 ± 0.7	>64

[a]IC_{50} values for inhibition of growth of the parasites *T. b. brucei* 427, *T. b. rhodesiense*, *Trypanosoma cruzi*, and *Leishmania infantum*, or for cytotoxicity toward human lung fibroblasts (MRC-5 cells). [b]Each value is the mean of 2 to 5 independent determinations (± standard deviation).

Experimental for the compounds of Table 6

Combustion analyses were performed by the Campbell Microanalytical Laboratory, University of Otago, Dunedin, New Zealand. Melting points were determined using an Electrothermal IA9100 melting point apparatus and are as read. NMR spectra were measured on a Bruker Avance 400 spectrometer at 400 MHz for ^{1}H and 100 MHz for ^{13}C and were referenced to Me_4Si or solvent resonances. Chemical shifts and coupling constants were recorded in units of ppm and hertz, respectively. High-resolution electrospray ionisation (HRESIMS) mass spectra were determined on a Bruker micrOTOF-Q II mass spectrometer. Low-resolution atmospheric pressure chemical ionisation (APCI) mass spectra were obtained for organic solutions using a ThermoFinnigan Surveyor MSQ mass spectrometer connected to a Gilson autosampler. Optical rotations were measured on a Schmidt + Haensch Polartronic NH8 polarimeter. Column chromatography was performed on silica gel (Merck 230-400 mesh). Thin-layer chromatography was carried out on aluminium-backed silica gel plates (Merck 60 F_{254}), with visualization of components by UV light (254 nm), I_2, or $KMnO_4$ staining. Tested compounds (including batches screened *in vivo*) were ≥95% pure, as determined by combustion analysis (results within 0.4% of theoretical values) and/or by HPLC conducted on an Agilent 1100 system, using a 150 mm x 3.2 mm Altima 5 µm reversed phase C18 column with diode array detection. Preparative SFC was performed by WuXi AppTec (Shanghai) Co., Ltd. on a Thar SFC Prep 80 instrument, using 250 mm x 30 mm CHIRALPAK AD-H or CHIRALCEL OD-H 5 µm preparative SFC columns, while chiral purity was assessed on an Agilent 1260 system, using the corresponding 250 mm x 4.6 mm 5 µm analytical SFC columns.

Synthesis of 12 and 38 (Scheme 1):

Procedure A: 2-Nitro-6-{[4-(trifluoromethoxy)benzyl]oxy}-6,7-dihydro-5*H*-imidazo[2,1-*b*][1,3]thiazine (30). A solution of 2-nitro-6,7-dihydro-5*H*-imidazo[2,1-*b*][1,3]thiazin-6-ol[1] (**42**) (500 mg, 2.49 mmol) and 4-(trifluoromethoxy)benzyl bromide (0.580 mL, 3.63 mmol) in anhydrous DMF (10 mL) under N_2 was treated with 60% NaH (152 mg, 3.80 mmol), then quickly degassed and resealed under N_2. After being stirred at 20 °C for 160 min, the mixture was cooled (CO_2/acetone), quenched with ice/aqueous $NaHCO_3$ (20 mL), added to brine (100 mL), and extracted with CH_2Cl_2 (6 x 100 mL). The combined extracts were evaporated to dryness under reduced pressure (at 30 °C) and the residue was chromatographed on silica gel. Elution with 0-30% EtOAc/petroleum ether first gave foreruns, and then further elution with 50% EtOAc/petroleum ether gave **30**[1] (863 mg, 93%) as a glassy yellow oil, which was used directly in the next step: ^{1}H NMR ($CDCl_3$) δ 7.66 (s, 1 H), 7.36 (br d, *J* = 8.7 Hz, 2 H), 7.22 (br d, *J* = 8.6 Hz, 2 H), 4.76 (d, *J* = 11.8 Hz, 1 H), 4.60 (d, *J* = 11.8 Hz, 1 H), 4.33-4.25 (m, 1 H), 4.18 (br dd, *J* = 13.6, 4.0 Hz, 1 H), 4.14 (br dd, *J* = 13.5, 5.4 Hz, 1 H), 3.35 (dd, *J* = 13.4, 3.0 Hz, 1 H), 3.31 (br dd, *J* = 13.4, 6.2 Hz, 1 H); APCI MS calcd for $C_{14}H_{13}F_3N_3O_4S$ *m/z* $[M + H]^+$ 376.1, found 376.1.

Procedure B: 2-Nitro-6-{[4-(trifluoromethoxy)benzyl]oxy}-6,7-dihydro-5*H*-imidazo[2,1-*b*][1,3]thiazine 8-oxide (12 and 38). 3-Chloroperoxybenzoic acid (573 mg of 70%, 2.32 mmol) was added to a precooled mixture of thiazine **30** (863 mg, 2.30 mmol) and powdered disodium hydrogen phosphate (720 mg, 5.07 mmol) in CH_2Cl_2 (60 mL) at -10 °C. After being stirred at -10 to 20 °C for 19 h (melting salt-ice bath), the mixture was added to an ice-cold aqueous solution of sodium sulfite (100 mL of 10%) and extracted with CH_2Cl_2 (4 x 100 mL). The extracts were sequentially washed with dilute aqueous $NaHCO_3$ (100 mL) and then the combined extracts were evaporated to dryness under reduced pressure (at 30 °C) and the remaining oil was chromatographed on silica gel. Elution with 0-50% EtOAc/petroleum ether

gave foreruns, then elution with 67% EtOAc/petroleum ether first gave a mixture (0.17 g) and then pure **12**[1] (495 mg, 55%) as a white foam: ^{1}H NMR ($CDCl_3$) δ 7.83 (s, 1 H), 7.37 (br d, *J* = 8.7 Hz, 2 H), 7.24 (br d, *J* = 8.7 Hz, 2 H), 4.89-4.81 (m, 1 H), 4.77 (d, *J* = 11.8 Hz, 1 H), 4.66 (d, *J* = 11.8 Hz, 1 H), 4.56 (br dd, *J* = 13.1, 4.6 Hz, 1 H), 4.15 (dd, *J* = 13.0, 8.6 Hz, 1 H), 3.66 (br dd, *J* = 13.9, 2.6 Hz, 1 H), 3.28 (dd, *J* = 13.9, 9.9 Hz, 1 H); APCI MS calcd for $C_{14}H_{13}F_3N_3O_5S$ *m/z* $[M + H]^+$ 392.1, found 392.1.

Further elution of the above column with 67% EtOAc/petroleum ether gave a mixture (68 mg) and then elution with EtOAc gave **38** (143 mg, 16%) as a white foam: ^{1}H NMR ($CDCl_3$) δ 7.85 (s, 1 H), 7.38 (br d, *J* = 8.7 Hz, 2 H), 7.20 (br d, *J* = 8.8 Hz, 2 H), 4.90 (d, *J* = 11.4 Hz, 1 H), 4.58 (ddd, *J* = 13.4, 4.4, 1.1 Hz, 1 H), 4.57 (d, *J* = 11.5 Hz, 1 H), 4.50-4.44 (m, 1 H), 4.26 (dd, *J* = 13.4, 3.3 Hz, 1 H), 3.84 (ddd, *J* = 15.0, 5.8, 1.1 Hz, 1 H), 3.36 (dd, *J* = 15.0, 2.3 Hz, 1 H); HPLC purity: 98.2%. Anal. ($C_{14}H_{12}F_3N_3O_5S$) C, H, N.

The above mixtures were individually purified by repeat chromatography on silica gel. In the first case, elution with CH_2Cl_2 initially gave foreruns, and then further elution with 0.33-0.5% MeOH/CH_2Cl_2 gave the sulfone **19**[1] (12 mg, 1.3%) as a white solid: mp 158-160 °C (lit.[1] mp 161 °C); ^{1}H NMR [$(CD_3)_2SO$] δ 8.61 (s, 1 H), 7.44 (br d, *J* = 8.7 Hz, 2 H), 7.34 (br d, *J* = 7.9 Hz, 2 H), 4.78 (d, *J* = 11.7 Hz, 1 H), 4.66-4.61 (m, 1 H), 4.61 (d, *J* = 11.7 Hz, 1 H), 4.60-4.50 (m, 2 H), 4.45 (dd, *J* = 14.4, 6.4 Hz, 1 H), 4.17 (dd, *J* = 14.4, 1.9 Hz, 1 H); APCI MS calcd for $C_{14}H_{13}F_3N_3O_6S$ *m/z* $[M + H]^+$ 408.0, found 408.1; HPLC purity: 98.4%.

Further elution of the above second column with 0.5-0.8% MeOH/CH_2Cl_2 gave **12** (151 mg, 17%). In the second case, successive chromatography using both solvent systems gave additional **12** (30 mg, 3%) and **38** (36 mg, 4%).

Synthesis of 24-27 (Scheme 1):

(6*S*)-2-Nitro-6-{[4-(trifluoromethoxy)benzyl]oxy}-6,7-dihydro-5*H*-imidazo[2,1-*b*][1,3]thiazine 8-oxide (24) and (6*R*)-2-nitro-6-{[4-(trifluoromethoxy)benzyl]oxy}-6,7-dihydro-5*H*-imidazo[2,1-*b*][1,3]thiazine 8-oxide (26). Thiazine oxide **12** (670 mg) was separated into its pure enantiomers by preparative chiral SFC, using a CHIRALPAK AD-H SFC column and an isocratic solvent system of 40% MeOH in CO_2 at a flow rate of 45 g/min, to first give **26** (256 mg, 38%) as a cream solid: mp 103-105 °C; ^{1}H NMR ($CDCl_3$) δ 7.84 (s, 1 H), 7.37 (br d, *J* = 8.6 Hz, 2 H), 7.24 (br d, *J* = 8.3 Hz, 2 H), 4.89-4.80 (m, 1 H), 4.77 (d, *J* = 11.8 Hz, 1 H), 4.66 (d, *J* = 11.8 Hz, 1 H), 4.56 (br dd, *J* = 13.0, 4.6 Hz, 1 H), 4.16 (dd, *J* = 13.0, 8.5 Hz, 1 H), 3.66 (br dd, *J* = 13.9, 2.1 Hz, 1 H), 3.29 (dd, *J* = 13.9, 9.9 Hz, 1 H); $[\alpha]^{26}_D$ 94.5 (*c* 1.005, $CHCl_3$); $[\alpha]^{26}_D$ 136.9 (*c* 1.001, MeOH); HPLC purity: 99.8%. Anal. ($C_{14}H_{12}F_3N_3O_5S$) C, H, N.

Further elution of the SFC column gave **24**[2] (245 mg, 37%) as a cream solid: mp 99-101 °C; ^{1}H NMR ($CDCl_3$) δ 7.84 (s, 1 H), 7.37 (br d, *J* = 8.6 Hz, 2 H), 7.24 (br d, *J* = 8.4 Hz, 2 H), 4.89-4.80 (m, 1 H), 4.77 (d, *J* = 11.8 Hz, 1 H), 4.66 (d, *J* = 11.8 Hz, 1 H), 4.56 (br dd, *J* = 13.0, 4.6 Hz, 1 H), 4.16 (dd, *J* = 13.0, 8.5 Hz, 1 H), 3.66 (br dd, *J* = 13.9, 2.1 Hz, 1 H), 3.29 (dd, *J* = 13.9, 9.9 Hz, 1 H); ^{1}H NMR (CD_3OD) δ 8.30 (s, 1 H), 7.46 (br d, *J* = 8.7 Hz, 2 H), 7.26 (br d, *J* = 8.0 Hz, 2 H), 4.79 (d, *J* = 11.9 Hz, 1 H), 4.76-4.65 (m, 3 H), 4.36-4.25 (m, 1 H), 3.83 (br dd, *J* = 13.6, 9.0 Hz, 1 H), 3.68 (br dd, *J* = 13.6, 2.0 Hz, 1 H); ^{13}C NMR (CD_3OD) δ 150.4 (q, J_{C-F} = 2.0 Hz), 149.2, 143.8, 138.1, 130.7 (2 C), 124.1, 122.3 (2 C), 122.1 (q, J_{C-F} = 255.5 Hz), 71.8, 68.7, 51.6, 51.3; $[\alpha]^{26}_D$ -93.8 (*c* 1.002, $CHCl_3$); $[\alpha]^{26}_D$ -134.5 (*c* 1.004, MeOH) {lit.[2] $[\alpha]^{20}_D$ -112.9 (*c* 0.82, MeOH)}; HPLC purity: 99.7%. Anal. ($C_{14}H_{12}F_3N_3O_5S$) C, H, N.

Analysis by chiral SFC [using a CHIRALPAK AD-H analytical SFC column and eluting with a gradient of 5-40% EtOH (containing 0.05% diethylamine) in CO_2 at a flow rate of 2.35 mL/min] determined that the ee's of **24** and **26** were both >99%.

(6*S*)-2-Nitro-6-{[4-(trifluoromethoxy)benzyl]oxy}-6,7-dihydro-5*H*-imidazo[2,1-*b*][1,3]thiazine 8-oxide (25) and (6*R*)-2-nitro-6-{[4-(trifluoromethoxy)benzyl]oxy}-6,7-dihydro-5*H*-imidazo[2,1-*b*][1,3]thiazine 8-oxide (27). Thiazine oxide **38** (162 mg) was separated into its pure enantiomers by preparative chiral SFC, using a CHIRALCEL OD-H preparative SFC column and an isocratic solvent system of 40% MeOH in CO_2 at a flow rate of 50 g/min, to first give **25** (33 mg, 20%) as a cream solid: mp 116-118 °C; ^{1}H NMR ($CDCl_3$) δ 7.85 (s, 1 H), 7.38 (br d, *J* = 8.6 Hz, 2 H), 7.19 (br d, *J* = 7.9 Hz, 2 H), 4.89 (d, *J* = 11.5 Hz, 1 H), 4.62-4.53 (m, 2 H), 4.51-4.45 (m, 1 H), 4.28 (dd, *J* = 13.4, 3.2 Hz, 1 H), 3.83 (ddd, *J* = 15.0, 5.8, 1.1 Hz, 1 H), 3.40 (dd, *J* = 15.0, 2.2 Hz, 1 H); $[\alpha]^{27}_D$ 123.0 (*c* 1.008, DMF); HRESIMS calcd for $C_{14}H_{12}F_3N_3NaO_5S$ *m*/*z* $[M + Na]^+$ 414.0342, found 414.0345; HPLC purity: 99.0%.

Further elution of the SFC column gave **27** (29 mg, 18%) as a cream solid: mp 111-112 °C; ^{1}H NMR ($CDCl_3$) δ 7.85 (s, 1 H), 7.38 (br d, *J* = 8.6 Hz, 2 H), 7.19 (br d, *J* = 8.2 Hz, 2 H), 4.89 (d, *J* = 11.5 Hz, 1 H), 4.62-4.53 (m, 2 H), 4.51-4.45 (m, 1 H), 4.29 (dd, *J* = 13.4, 3.2 Hz, 1 H), 3.83 (br dd, *J* = 15.0, 5.7 Hz, 1 H), 3.40 (dd, *J* = 15.0, 2.2 Hz, 1 H); $[\alpha]^{26}_D$ -121.9 (*c* 1.001, $CHCl_3$); $[\alpha]^{27}_D$ -119.6 (*c* 0.953, DMF); HRESIMS calcd for $C_{14}H_{12}F_3N_3NaO_5S$ *m*/*z* $[M + Na]^+$ 414.0342, found 414.0339; HPLC purity: 99.8%.

Analysis by chiral SFC [using a CHIRALCEL OD-H analytical SFC column and eluting with a gradient of 5-40% EtOH (containing 0.05% diethylamine) in CO_2 at a flow rate of 2.35 mL/min] determined that the ee of **25** was 98.9% and the ee of **27** was 97.2%.

Synthesis of 28 (Scheme 2A):

1-(Benzyloxy)-3-[(triisopropylsilyl)oxy]propan-2-ol (44). Chlorotriisopropylsilane (3.90 mL, 18.2 mmol) was slowly added to a solution of 3-(benzyloxy)propane-1,2-diol (**43**) (3.00 g, 16.5 mmol) and imidazole (2.48 g, 36.4 mmol) in anhydrous DMF 40 mL) under N_2. After being stirred at 20 °C for 3 d, the resulting mixture was added to ice-water (200 mL) and extracted with 50% EtOAc/petroleum ether (2 x 200 mL). The extracts were washed with water (200 mL) and then concentrated under reduced pressure (at 30 °C) and the remaining oil was chromatographed on silica gel. Elution with 0-10% CH_2Cl_2/petroleum ether first gave foreruns, and then further elution with 25% Et_2O/petroleum ether gave **44**[3] (5.11 g, 92%) as a colourless oil; ^{1}H NMR ($CDCl_3$) δ 7.39-7.24 (m, 5 H), 4.56 (s, 2 H), 3.93-3.83 (m, 1 H), 3.77 (dd, *J* = 9.8, 4.9 Hz, 1 H), 3.74 (dd, *J* = 9.8, 5.8 Hz, 1 H), 3.57 (dd, *J* = 9.6, 5.1 Hz, 1 H), 3.53 (dd, *J* = 9.6, 5.8 Hz, 1 H), 2.54 (br d, *J* = 5.1 Hz, 1 H), 1.17-0.99 (m, 21 H); HRESIMS calcd for $C_{19}H_{34}NaO_3Si$ *m*/*z* $[M + Na]^+$ 361.2169, found 361.2173.

{3-(Benzyloxy)-2-[4-(trifluoromethoxy)phenoxy]propoxy}triisopropylsilane (45). Diethyl azodicarboxylate (3.20 mL, 20.6 mmol) was added dropwise to a stirred solution of alcohol **44** (5.11 g, 15.1 mmol), 4-(trifluoromethoxy)phenol (2.65 mL, 20.5 mmol) and triphenylphosphine (5.15 g, 19.6 mmol) in anhydrous THF (15 mL) under N_2 at 0 °C. After being stirred at 20 °C for 4.5 d, the mixture was concentrated under reduced pressure to give an oil, which was chromatographed on silica gel. Elution with petroleum ether first gave foreruns, and then further elution with 0-1% Et_2O/petroleum ether gave **45** (5.67 g, 75%) as a colourless oil; ^{1}H NMR ($CDCl_3$) δ 7.37-7.24 (m, 5 H), 7.10 (br d, *J* = 9.1 Hz, 2 H), 6.96 (br d, *J* = 9.2 Hz, 2 H), 4.57 (s, 2 H), 4.48-4.41 (m, 1 H), 3.92 (dd, *J* = 10.5, 5.3 Hz, 1 H), 3.89

(dd, J = 10.5, 5.4 Hz, 1 H), 3.77 (dd, J = 10.3, 4.4 Hz, 1 H), 3.69 (dd, J = 10.4, 5.4 Hz, 1 H), 1.15-0.96 (m, 21 H); HRESIMS calcd for $C_{26}H_{37}F_3NaO_4Si$ m/z $[M + Na]^+$ 521.2305, found 521.2294.

2-[4-(Trifluoromethoxy)phenoxy]-3-[(triisopropylsilyl)oxy]propan-1-ol (46). A mixture of benzyl ether **45** (5.67 g, 11.4 mmol) and 10% palladium on activated carbon (570 mg) in EtOAc (80 mL) and EtOH (80 mL) was hydrogenated at 60 psi for 48 h. The resulting mixture was filtered through Celite, washing with 33% MeOH/CH_2Cl_2, the filtrate was evaporated to dryness under reduced pressure, and the residue was chromatographed on silica gel. Elution with 0-2% Et_2O/petroleum ether first gave foreruns, and then further elution with 2-25% Et_2O/petroleum ether gave **46** (4.55 g, 98%) as a colourless oil; ^1H NMR ($CDCl_3$) δ 7.13 (br d, J = 9.1 Hz, 2 H), 6.96 (br d, J = 9.2 Hz, 2 H), 4.43-4.36 (m, 1 H), 3.99-3.82 (m, 4 H), 2.13 (t, J = 6.4 Hz, 1 H), 1.18-0.98 (m, 21 H); HRESIMS calcd for $C_{19}H_{31}F_3NaO_4Si$ m/z $[M + Na]^+$ 431.1836, found 431.1821.

{3-Iodo-2-[4-(trifluoromethoxy)phenoxy]propoxy}triisopropylsilane (47). A solution of iodine (3.85 g, 15.2 mmol) in anhydrous CH_2Cl_2 (8 x 10 mL, then 2 x 10 mL to rinse) was added dropwise over 1 h (with water bath cooling) to a mixture of alcohol **46** (4.55 g, 11.1 mmol), imidazole (1.97 g, 28.9 mmol) and triphenylphosphine (3.81 g, 14.5 mmol) in anhydrous CH_2Cl_2 (40 mL) under N_2. After being stirred at 20 °C for 15 h, the mixture was concentrated under reduced pressure, and the residual oil was added to excess petroleum ether (300 mL) at the top of a silica gel column (80 g in petroleum ether), rinsing on with CH_2Cl_2 (5 x 3 mL). Elution with 0-5% Et_2O/petroleum ether gave **47** (5.67 g, 98%) as a colourless oil; ^1H NMR ($CDCl_3$) δ 7.14 (br d, J = 9.1 Hz, 2 H), 6.95 (br d, J = 9.1 Hz, 2 H), 4.26-4.17 (m, 1 H), 3.99 (dd, J = 10.5, 4.8 Hz, 1 H), 3.90 (dd, J = 10.5, 5.5 Hz, 1 H), 3.51 (dd, J = 10.5, 5.5 Hz, 1 H), 3.40 (dd, J = 10.5, 4.8 Hz, 1 H), 1.18-1.00 (m, 21 H); HRESIMS calcd for $C_{19}H_{30}F_3INaO_3Si$ m/z $[M + Na]^+$ 541.0853, found 541.0848.

2-Chloro-4-nitro-1-{2-[4-(trifluoromethoxy)phenoxy]-3-[(triisopropylsilyl)oxy]propyl}-1*H*-imidazole (48). A mixture of iodide **47** (3.50 g, 6.75 mmol), 2-chloro-4-nitro-1*H*-imidazole (1.07 g, 7.25 mmol) and powdered K_2CO_3 (1.08 g, 7.81 mmol) in anhydrous DMF (12.5 mL) under N_2 was stirred at 85 °C for 64 h. The resulting cooled mixture was added to aq $NaHCO_3$ (100 mL) and extracted with CH_2Cl_2 (6 x 100 mL), and then the combined extracts were evaporated to dryness under reduced pressure (at 30 °C) and the remaining oil was chromatographed on silica gel. Elution with 0-10% Et_2O/petroleum ether first gave foreruns, and then further elution with 10-20% Et_2O/petroleum ether gave **48** (3.20 g, 88%) as a yellow oil; ^1H NMR ($CDCl_3$) δ 7.88 (s, 1 H), 7.14 (br d, J = 9.1 Hz, 2 H), 6.83 (br d, J = 9.2 Hz, 2 H), 4.56 (dd, J = 14.2, 3.1 Hz, 1 H), 4.55-4.47 (m, 1 H), 4.32 (dd, J = 14.2, 7.3 Hz, 1 H), 3.96 (dd, J = 10.8, 4.0 Hz, 1 H), 3.80 (dd, J = 10.8, 6.8 Hz, 1 H), 1.19-1.00 (m, 21 H); HRESIMS calcd for $C_{22}H_{31}ClF_3N_3NaO_5Si$ m/z $[M + Na]^+$ 562.1546, 560.1566, found 562.1551, 560.1575.

3-(2-Chloro-4-nitro-1*H*-imidazol-1-yl)-2-[4-(trifluoromethoxy)phenoxy]propan-1-ol (49). A solution of silyl ether **48** (3.20 g, 5.95 mmol) in anhydrous THF (80 mL) under N_2 at 0 °C was treated with TBAF (6.5 mL of a 1 M solution in THF, 6.50 mmol). The mixture was stirred at 0-5 °C for 5 h, and then added to ice/aqueous $NaHCO_3$ (100 mL) and extracted with EtOAc (50 mL, then 4 x 100 mL). The combined extracts were evaporated to dryness under reduced pressure and the residue was chromatographed on silica gel. Elution with 0-50% Et_2O/petroleum ether first gave foreruns, and then further elution with 67% Et_2O/petroleum ether gave **49** (1.89 g, 83%) as a yellow oil; ^1H NMR ($CDCl_3$) δ 7.88 (s, 1 H), 7.16 (br d, J = 9.1 Hz, 2 H), 6.87 (br d, J = 9.2 Hz, 2 H), 4.62-4.54 (m, 1 H), 4.47 (dd, J = 14.7, 4.1 Hz, 1

H), 4.41 (dd, J = 14.7, 7.2 Hz, 1 H), 3.95-3.80 (m, 2 H), 1.92 (br t, J = 5.1 Hz, 1 H); HRESIMS calcd for $C_{13}H_{11}ClF_3N_3NaO_5$ *m/z* [M + Na]$^+$ 406.0207, 404.0232, found 406.0221, 404.0246.

3-(2-Chloro-4-nitro-1*H*-imidazol-1-yl)-2-[4-(trifluoromethoxy)phenoxy]propyl 4-methylbenzenesulfonate (50) and 2-chloro-1-{3-chloro-2-[4-(trifluoromethoxy)phenoxy]-propyl}-4-nitro-1*H*-imidazole (51). A solution of tosyl chloride (1.44 g, 7.55 mmol) in anhydrous pyridine (5 mL, then 2 x 2.5 mL to rinse) was added dropwise to a stirred solution of alcohol **49** (1.88 g, 4.93 mmol) in anhydrous pyridine (10 mL) under N_2 at 0 °C. The mixture was stirred at 0 to 20 °C for 25 h and then added to ice-water (100 mL) and extracted with CH_2Cl_2 (4 x 100 mL). The combined extracts were concentrated to dryness under reduced pressure (at 30 °C) and the remaining oil was chromatographed on silica gel. Elution with CH_2Cl_2 first gave impure **51** (0.19 g), which was chromatographed again on silica gel, eluting with 50% Et_2O/petroleum ether (foreruns) and then with CH_2Cl_2, to give **51** (176 mg, 9%) as a cream solid (following trituration in cold pentane): mp 86-87 °C; ^{1}H NMR ($CDCl_3$) δ 7.89 (s, 1 H), 7.18 (br d, J = 9.1 Hz, 2 H), 6.87 (br d, J = 9.1 Hz, 2 H), 4.68-4.60 (m, 1 H), 4.58 (dd, J = 14.7, 3.0 Hz, 1 H), 4.41 (dd, J = 14.7, 7.7 Hz, 1 H), 3.74 (dd, J = 12.1, 3.4 Hz, 1 H), 3.60 (dd, J = 12.1, 7.5 Hz, 1 H); HRESIMS calcd for $C_{13}H_{10}Cl_2F_3N_3NaO_4$ *m/z* [M + Na]$^+$ 425.9842, 423.9865, 421.9893, found 425.9840, 423.9868, 421.9897.

Further elution of the initial column above with CH_2Cl_2 and 20% Et_2O/CH_2Cl_2 gave **50** (2.23 g, 84%) as a yellow oil; ^{1}H NMR ($CDCl_3$) δ 7.78 (s, 1 H), 7.77 (br d, J = 8.4 Hz, 2 H), 7.36 (br d, J = 8.0 Hz, 2 H), 7.12 (br d, J = 9.0 Hz, 2 H), 6.77 (br d, J = 9.2 Hz, 2 H), 4.71-4.63 (m, 1 H), 4.46 (dd, J = 14.9, 3.3 Hz, 1 H), 4.35 (dd, J = 14.9, 7.5 Hz, 1 H), 4.25 (dd, J = 11.4, 3.8 Hz, 1 H), 4.07 (dd, J = 11.4, 6.5 Hz, 1 H), 2.46 (s, 3 H); HRESIMS calcd for $C_{20}H_{17}ClF_3N_3NaO_7S$ *m/z* [M + Na]$^+$ 560.0297, 558.0320, found 560.0307, 558.0326.

2-Nitro-6-[4-(trifluoromethoxy)phenoxy]-6,7-dihydro-5*H*-imidazo[2,1-*b*][1,3]thiazine (28). *n*-Butyllithium (1.80 mL of a 2.5 M solution in hexanes, 4.50 mmol) was added dropwise to a stirred solution of triisopropylsilanethiol (0.92 mL, 4.29 mmol) in anhydrous freshly distilled THF (20 mL) under N_2 at -5 °C. After 10 min, this mixture was added dropwise to a stirred solution of tosylate **50** (2.23 g, 4.16 mmol) in anhydrous freshly distilled THF (40 mL) under N_2 at -78 °C, rinsing in residues with additional anhydrous THF (2 x 5 mL). Following further stirring at 20 °C for 48 h, the resulting mixture was added to ice/aqueous $NaHCO_3$ (100 mL) and extracted with EtOAc (3 x 100 mL). The combined extracts were evaporated to dryness under reduced pressure (at 30 °C) and the residue was redissolved in anhydrous freshly distilled THF (40 mL) under N_2. TBAF (9.0 mL of a 1 M solution in THF, 9.0 mmol) was added and the solution was stirred at 20 °C for 13 h, then added to ice/aqueous $NaHCO_3$ (100 mL), and extracted with EtOAc (4 x 100 mL). The combined extracts were evaporated to dryness under reduced pressure (at 30 °C) and the remaining oil was chromatographed on silica gel. Elution with 0-33% EtOAc/petroleum ether first gave foreruns, and then further elution with 50% EtOAc/petroleum ether gave impure product, which was chromatographed twice more on silica gel. Elution with Et_2O or CH_2Cl_2 first gave foreruns, and then further elution with 10% Et_2O/CH_2Cl_2 gave **28** (460 mg, 31%) as a pale yellow-orange solid: mp (CH_2Cl_2/pentane) 147-150 °C; ^{1}H NMR [$(CD_3)_2SO$] δ 8.43 (s, 1 H), 7.33 (br d, J = 9.1 Hz, 2 H), 7.17 (br d, J = 9.2 Hz, 2 H), 5.42-5.32 (m, 1 H), 4.46 (ddd, J = 14.2, 3.6, 1.4 Hz, 1 H), 4.39 (dd, J = 14.1, 2.8 Hz, 1 H), 3.64 (dd, J = 13.5, 1.7 Hz, 1 H), 3.49 (ddd, J = 13.5, 5.3, 1.5 Hz, 1 H); ^{13}C NMR [$(CD_3)_2SO$] δ 154.6, 146.1, 142.7 (q, J_{C-F} = 1.6 Hz), 138.7, 123.4, 122.7 (2 C), 120.1 (q, J_{C-F} = 255.5 Hz), 117.7 (2 C), 65.7, 48.9, 27.9; HPLC purity: 99.7%. Anal. ($C_{13}H_{10}F_3N_3O_4S$) C, H, N.

Synthesis of 33, 36 and 40 (Scheme 2A):

2-Nitro-6-[4-(trifluoromethoxy)phenoxy]-6,7-dihydro-5*H*-imidazo[2,1-*b*][1,3]thiazine 8-oxide (33 and 36) and 2-nitro-6-[4-(trifluoromethoxy)phenoxy]-6,7-dihydro-5*H*-imidazo[2,1-*b*][1,3]thiazine 8,8-dioxide (40). Reaction of thiazine **28** with *m*-CPBA (1.18 equiv) and disodium hydrogen phosphate, using procedure B for 23 h, followed by chromatography of the product on silica gel, eluting with 0-1% EtOAc/CH_2Cl_2 (foreruns) and then with 1-2% EtOAc/CH_2Cl_2, first gave **40** (11%) as a white solid: mp (CH_2Cl_2/pentane) 172-174 °C; ^{1}H NMR [$(CD_3)_2SO$] δ 8.69 (s, 1 H), 7.36 (br d, J = 9.1 Hz, 2 H), 7.16 (br d, J = 9.2 Hz, 2 H), 5.63-5.54 (m, 1 H), 4.72 (dd, J = 14.1, 2.9 Hz, 1 H), 4.67 (dd, J = 14.1, 4.1 Hz, 1 H), 4.35 (dd, J = 14.6, 5.9 Hz, 1 H), 4.29 (dd, J = 14.5, 2.6 Hz, 1 H); ^{13}C NMR [$(CD_3)_2SO$] δ 154.3, 145.8, 143.0 (q, $J_{C\text{-}F}$ = 1.6 Hz), 141.5, 123.1, 122.7 (2 C), 120.1 (q, $J_{C\text{-}F}$ = 255.5 Hz), 117.7 (2 C), 69.1, 53.5, 49.9; HPLC purity: 98.9%. Anal. ($C_{13}H_{10}F_3N_3O_6S$) C, H, N.

Further elution of the above column with 3-4% EtOAc/CH_2Cl_2 gave **33** (82%) as a white solid: mp (CH_2Cl_2/pentane) 171-172 °C; ^{1}H NMR [$(CD_3)_2SO$] δ 8.67 (s, 1 H), 7.37 (br d, J = 8.4 Hz, 2 H), 7.20 (br d, J = 9.2 Hz, 2 H), 5.62-5.51 (m, 1 H), 4.81 (dd, J = 13.1, 3.9 Hz, 1 H), 4.34 (dd, J = 13.1, 7.7 Hz, 1 H), 3.96 (dd, J = 13.6, 9.4 Hz, 1 H), 3.80 (dd, J = 13.6, 1.4 Hz, 1 H); ^{13}C NMR [$(CD_3)_2SO$] δ 154.7, 146.8, 142.9 (q, $J_{C\text{-}F}$ = 1.6 Hz), 142.8, 123.8, 122.9 (2 C), 120.1 (q, $J_{C\text{-}F}$ = 255.6 Hz), 117.4 (2 C), 66.4, 49.3, 48.9; HPLC purity: 99.7%. Anal. ($C_{13}H_{10}F_3N_3O_5S$) C, H, N.

Further elution of the above column with 10% MeOH/CH_2Cl_2 gave **36** (2%) as a cream solid: mp (MeOH/CH_2Cl_2/hexane) 229-232 °C; ^{1}H NMR [$(CD_3)_2SO$] δ 8.69 (s, 1 H), 7.32 (br d, J = 8.4 Hz, 2 H), 7.13 (br d, J = 9.2 Hz, 2 H), 5.55-5.47 (m, 1 H), 4.76 (dd, J = 14.2, 2.5 Hz, 1 H), 4.38 (dd, J = 14.2, 2.6 Hz, 1 H), 3.78 (ddd, J = 15.2, 4.5, 1.0 Hz, 1 H), 3.73 (dd, J = 15.2, 2.5 Hz, 1 H); HRESIMS calcd for $C_{13}H_{11}F_3N_3O_5S$ *m*/*z* $[M + H]^+$ 378.0366, found 378.0371; HPLC purity: 99.5%.

Synthesis of 29 (Scheme 2B):

2-Nitro-6-{[5-(trifluoromethyl)pyridin-2-yl]oxy}-6,7-dihydro-5*H*-imidazo[2,1-*b*][1,3]thiazine (29). Reaction of alcohol **42**[1] with 2-chloro-5-(trifluoromethyl)pyridine (**52**) (3.0 equiv) and NaH (1.6 equiv), using procedure A at 0-20 °C for 3.5 h, followed by chromatography of the product on silica gel, eluting with CH_2Cl_2 (foreruns) and then with 1% EtOAc/CH_2Cl_2, gave **29** (69%) as a yellow-orange solid: mp (Et_2O/pentane) 88-90 °C; ^{1}H NMR ($CDCl_3$) δ 8.48-8.43 (m, 1 H), 7.87 (br dd, J = 8.8, 2.4 Hz, 1 H), 7.73 (s, 1 H), 6.90 (br d, J = 8.7 Hz, 1 H), 5.97-5.90 (m, 1 H), 4.44 (br dd, J = 13.6, 4.4 Hz, 1 H), 4.39 (dd, J = 13.7, 3.7 Hz, 1 H), 3.58 (ddd, J = 13.5, 5.3, 0.9 Hz, 1 H), 3.54 (dd, J = 13.4, 3.2 Hz, 1 H); HPLC purity: 99.3%. Anal. ($C_{12}H_9F_3N_4O_3S$) C, H, N.

Synthesis of 34, 37 and 41 (Scheme 2B):

2-Nitro-6-{[5-(trifluoromethyl)pyridin-2-yl]oxy}-6,7-dihydro-5*H*-imidazo[2,1-*b*][1,3]thiazine 8-oxide (34 and 37) and 2-nitro-6-{[5-(trifluoromethyl)pyridin-2-yl]oxy}-6,7-dihydro-5*H*-imidazo[2,1-*b*][1,3]thiazine 8,8-dioxide (41). Reaction of thiazine **29** with *m*-CPBA (1.33 equiv) and disodium hydrogen phosphate, using procedure B for 25 h, followed by chromatography of the product on silica gel, eluting with CH_2Cl_2 (foreruns) and then with 0.25% MeOH/CH_2Cl_2, first gave **41** (28%) as a white solid: mp (CH_2Cl_2/hexane) 193-196 °C; ^{1}H NMR [$(CD_3)_2SO$] δ 8.70 (s, 1 H), 8.70-8.66 (m, 1 H), 8.14 (br dd, J = 8.8, 2.4 Hz, 1 H), 7.03 (br d, J = 8.8 Hz, 1 H), 6.17-6.08 (m, 1 H), 4.77 (d, J = 3.3 Hz, 2 H), 4.46

(dd, J = 14.5, 6.1 Hz, 1 H), 4.35 (dd, J = 14.5, 2.2 Hz, 1 H); HPLC purity: 99.4%. Anal. ($C_{12}H_9F_3N_4O_5S$) C, H, N.

Further elution of the above column with 0.25-0.33% MeOH/CH_2Cl_2 gave **34** (60%) as a white solid: mp (CH_2Cl_2/hexane) 217-219 °C; ^{1}H NMR ($CDCl_3$) δ 8.51-8.46 (m, 1 H), 7.90 (s, 1 H), 7.89 (br dd, J = 8.7, 2.2 Hz, 1 H), 6.87 (br d, J = 8.7 Hz, 1 H), 6.42-6.34 (m, 1 H), 4.98 (dd, J = 13.3, 4.3 Hz, 1 H), 4.42 (dd, J = 13.3, 6.9 Hz, 1 H), 3.86 (br dd, J = 14.1, 4.1 Hz, 1 H), 3.59 (dd, J = 14.0, 8.7 Hz, 1 H); HPLC purity: 99.3%. Anal. ($C_{12}H_9F_3N_4O_4S$) C, H, N.

Further elution of the above column with 0.33-1% MeOH/CH_2Cl_2 gave **37** (11%) as a cream solid: mp (Et_2O/pentane) 113 °C (dec); ^{1}H NMR ($CDCl_3$) δ 8.48-8.42 (m, 1 H), 7.96 (s, 1 H), 7.86 (br dd, J = 8.8, 2.3 Hz, 1 H), 6.91 (br d, J = 8.7 Hz, 1 H), 6.14-6.06 (m, 1 H), 4.84 (ddd, J = 13.8, 4.2, 1.0 Hz, 1 H), 4.54 (dd, J = 13.8, 3.6 Hz, 1 H), 4.09 (ddd, J = 15.1, 5.5, 1.1 Hz, 1 H), 3.47 (dd, J = 15.1, 2.8 Hz, 1 H); HRESIMS calcd for $C_{12}H_9F_3N_4NaO_4S$ m/z $[M + Na]^+$ 385.0189, found 385.0198; HPLC purity: 98.7%.

Synthesis of 32 (Scheme 2C):

6-[(5-Bromopyridin-2-yl)methoxy]-2-nitro-6,7-dihydro-5*H*-imidazo[2,1-*b*][1,3]thiazine (54). Reaction of alcohol **42**[1] with 5-bromo-2-(bromomethyl)pyridine[4] (**53**) (1.3 equiv) and NaH (1.6 equiv), using procedure A at 0-20 °C for 3 h, followed by chromatography of the product on silica gel, eluting with 0-0.5% MeOH/CH_2Cl_2 (foreruns) and then with 0.5-0.75% MeOH/CH_2Cl_2, gave **54** (79%) as a yellow solid: mp (MeOH/CH_2Cl_2/hexane) 180-181 °C; ^{1}H NMR [$(CD_3)_2SO$] δ 8.65 (br d, J = 2.3 Hz, 1 H), 8.36 (s, 1 H), 8.05 (dd, J = 8.4, 2.4 Hz, 1 H), 7.35 (br d, J = 8.5 Hz, 1 H), 4.75 (d, J = 13.3 Hz, 1 H), 4.66 (d, J = 13.3 Hz, 1 H), 4.49-4.43 (m, 1 H), 4.39 (dd, J = 13.9, 3.8 Hz, 1 H), 4.24 (dd, J = 13.8, 2.6 Hz, 1 H), 3.54 (d, J = 3.2 Hz, 2 H). Anal. ($C_{12}H_{11}BrN_4O_3S$) C, H, N.

2-Nitro-6-({5-[4-(trifluoromethoxy)phenyl]pyridin-2-yl}methoxy)-6,7-dihydro-5*H*-imidazo[2,1-*b*][1,3]thiazine (32). A stirred mixture of bromide **54** (100 mg, 0.269 mmol), 4-(trifluoromethoxy)phenylboronic acid (112 mg, 0.544 mmol) and Pd(dppf)Cl_2 (100 mg, 0.137 mmol) in DMF (2.5 mL), toluene (1.6 mL) and EtOH (1.2 mL) was degassed for 10 min (vacuum pump) and then N_2 was added. An aqueous solution of Na_2CO_3 (0.65 mL of 2 M, 1.30 mmol) was added by syringe, the stirred mixture was again degassed for 11 min, and then N_2 was added. The mixture was stirred at 84 °C for 3 h and then cooled. Additional Pd(dppf)Cl_2 (50.1 mg, 0.068 mmol) was added, the stirred mixture was again degassed for 1 min, and then N_2 was added. The resulting mixture was stirred at 84 °C for a further 1.5 h and then cooled, diluted with aqueous $NaHCO_3$ (50 mL), and extracted with CH_2Cl_2 (6 x 50 mL). The combined extracts were evaporated to dryness under reduced pressure (at 30 °C), and the residue was chromatographed on silica gel. Elution with 0-0.5% MeOH/CH_2Cl_2 first gave foreruns, and then further elution with 0.66-1% MeOH/CH_2Cl_2 gave **32** (54 mg, 44%) as a yellow-orange solid: mp (Et_2O/pentane) 122-123 °C; ^{1}H NMR ($CDCl_3$) δ 8.76 (br d, J = 2.3 Hz, 1 H), 7.87 (dd, J = 8.1, 2.3 Hz, 1 H), 7.65 (s, 1 H), 7.59 (br d, J = 8.8 Hz, 2 H), 7.46 (br d, J = 8.0 Hz, 1 H), 7.34 (br d, J = 8.8 Hz, 2 H), 4.89 (d, J = 12.8 Hz, 1 H), 4.79 (d, J = 12.8 Hz, 1 H), 4.48-4.42 (m, 1 H), 4.26 (dd, J = 13.3, 5.1 Hz, 1 H), 4.21 (dd, J = 13.4, 3.7 Hz, 1 H), 3.44-3.35 (m, 2 H); HPLC purity: 99.9%. Anal. ($C_{19}H_{15}F_3N_4O_4S$) C, H, N.

Synthesis of 35 and 39 (Scheme 2C):

2-Nitro-6-({5-[4-(trifluoromethoxy)phenyl]pyridin-2-yl}methoxy)-6,7-dihydro-5*H*-imidazo[2,1-*b*][1,3]thiazine 8-oxide (35 and 39). Reaction of thiazine **32** with *m*-CPBA (1.44 equiv) and disodium hydrogen phosphate, using procedure B for 18 h, and then further reaction with additional *m*-CPBA (0.28 equiv) for 34 h, followed by chromatography of the product on silica gel, eluting with 0-0.66% MeOH/CH_2Cl_2 (foreruns) and then with 0.66-1% MeOH/CH_2Cl_2, first gave **35** (55%) as a cream solid: mp (CH_2Cl_2/pentane) 130-131 °C; ^{1}H NMR ($CDCl_3$) δ 8.80 (d, *J* = 2.2 Hz, 1 H), 7.89 (dd, *J* = 8.0, 2.3 Hz, 1 H), 7.84 (s, 1 H), 7.60 (br d, *J* = 8.6 Hz, 2 H), 7.41 (d, *J* = 8.1 Hz, 1 H), 7.35 (br d, *J* = 8.5 Hz, 2 H), 5.00-4.90 (m, 1 H), 4.90 (d, *J* = 12.7 Hz, 1 H), 4.85 (d, *J* = 12.6 Hz, 1 H), 4.70 (dd, *J* = 13.2, 4.4 Hz, 1 H), 4.29 (dd, *J* = 13.1, 7.9 Hz, 1 H), 3.71 (dd, *J* = 13.9, 2.5 Hz, 1 H), 3.42 (dd, *J* = 13.9, 9.3 Hz, 1 H); HPLC purity: 99.1%. Anal. ($C_{19}H_{15}F_3N_4O_5S$) C, H, N.

Further elution of the above column with 1.5-3% MeOH/CH_2Cl_2 gave **39** (8%) as a cream solid: mp (CH_2Cl_2/pentane) 121-124 °C; ^{1}H NMR ($CDCl_3$) δ 8.74 (br d, *J* = 2.3 Hz, 1 H), 7.85 (dd, *J* = 8.0, 2.4 Hz, 1 H), 7.85 (s, 1 H), 7.58 (br d, *J* = 8.8 Hz, 2 H), 7.50 (br d, *J* = 8.1 Hz, 1 H), 7.33 (br d, *J* = 8.7 Hz, 2 H), 4.99 (d, *J* = 12.2 Hz, 1 H), 4.77 (d, *J* = 12.2 Hz, 1 H), 4.72-4.63 (m, 2 H), 4.34-4.26 (m, 1 H), 3.95-3.86 (m, 1 H), 3.43 (dd, *J* = 15.0, 2.2 Hz, 1 H); HRESIMS calcd for $C_{19}H_{16}F_3N_4O_5S$ *m*/*z* $[M + H]^+$ 469.0788, found 469.0781; HPLC purity: 99.5%.

***In Vitro* Parasite Growth Inhibition Assays.** Initial screening of library compounds (at 2 and 5 µg/mL) against the bloodstream form of *T. brucei* (427 strain), followup IC_{50} testing, and assessing for cytotoxicity toward L929 mouse fibroblast cells were carried out by Scynexis, Inc., Research Triangle Park, NC 27713, USA, as described.[5] Further assays quantifying the growth inhibitory action of compounds against four protozoan parasites (*T. brucei*, *T. rhodesiense, T. cruzi*, and *L. infantum*) and measuring any cytotoxic effects on human lung fibroblasts (MRC-5 cells) were conducted at the University of Antwerp (LMPH), as reported.[6]

Microsomal and Mouse S9 Stability Assays. Microsomal stability studies on compounds **24-27** and **35** were performed by WuXi AppTec (Shanghai) Co., Ltd., 288 FuTe ZhongLu, WaiGaoQiao Free Trade Zone, Shanghai 200131, China, via a reported method[7] (except that the concentration of microsomal protein was 0.4 mg/mL). The earlier compounds (**9-23**) were tested against a mouse liver S9 subcellular fraction (2.5 mg/mL protein) by Scynexis, Inc., Research Triangle Park, NC 27713, USA, according to a published procedure.[5]

Permeability Assays. The evaluation of compound **35** was performed by WuXi AppTec (Shanghai) Co., Ltd. MDCK-MDR1 cells were seeded onto polyethylene membranes in 96-well plates at 2 x 10^5 cells/mL, giving confluent cell monolayer formation over 4-7 d. A solution of **35** (2 µM in <1% DMSO/HBSS buffer) was applied to the apical or basolateral side of the cell monolayer. Permeation of the compound from A to B direction or B to A direction was determined in triplicate over a 150 min incubation at 37 °C and 5% CO_2 (95% humidity). In addition, the efflux ratio of **35** was also determined. Test and reference compounds (fenoterol, propranolol, digoxin) were quantified by LC-MS/MS analysis based on the peak area ratio of analyte/internal standard. The earlier compounds (**9-23**) were assessed by Scynexis, Inc., according to a published protocol,[5] involving the incubation of test substrates (3 µM) at 37 °C for 1 h with shaking (160 rpm), in the presence or absence of the P-gp inhibitor GF120918 (2 µM).

Solubility Determinations (ACSRC). The solid compound sample was mixed with water or 0.1 M HCl (enough to make a 2 mM solution) in an Eppendorf tube, and the suspension was

sonicated for 15 min and then centrifuged at 13000 rpm for 6 min. An aliquot of the clear supernatant was diluted 2-fold with water (or 0.1 M HCl), and then HPLC was implemented. The kinetic solubility was calculated by comparing the peak area obtained with that from a standard solution of the compound in DMSO (after allowing for varying dilution factors and injection volumes).

Mouse Pharmacokinetics. The in-life portion of PK testing was carried out by ViviSource Laboratories, Inc., Massachusetts, USA, and PK samples were analysed by Scynexis, Inc. Test compounds (**9**, **12**, **17**, and **19**) were each administered as single doses to groups of 10 male CD-1 mice by bolus intravenous injection or oral gavage. Intravenous dosing (at a nominal dose of 2 mg/kg) employed a solution vehicle comprising either 20% EtOH, 20% propylene glycol, and 30% PEG-400 in normal saline (for powders **9**, **17**, and **19**) or 0.5% (hydroxypropyl)methylcellulose, 0.4% Tween 80, 0.5% benzyl alcohol, and 20% DMSO in deionised water (for oil **12**). Oral dosing (at a nominal dose of 50 mg/kg) was as a suspension in 0.5% methylcellulose, 0.08% Tween 80 in water (except for oil **12**, where the formulation additionally contained 20% DMSO). Samples derived from plasma, whole blood, and brain tissue (at 0.25, 0.5, 1, 2, 4, 6, 8, 12, 18, and 24 h) were centrifuged prior to analysis by LC-MS/MS and the PK parameters were determined using non-compartmental analysis in Microsoft Excel.

Mouse Model for Acute HAT Infection. Compound **24** was tested for *in vivo* efficacy against acute HAT infection caused by *T. b. brucei* (EATRO 110 strain) at the Haskins Laboratories, Pace University. In this assay, **24** was given orally once daily for 4 days as a suspension formulation (0.8% CMC, 0.1% SDS in water) to groups of 4 or 5 infected female Swiss Webster mice, starting 24 h postinfection, according to a published protocol.[5] Animals were monitored daily for signs of infection and weekly for parasites in tail vein blood smears, starting 1 week after dosing ended. Infected animals or those showing signs of infection were euthanized. Untreated infected control animals died after 5-7 days; mice remaining parasite free for more than 30 days beyond the end of the treatment period were considered cured.

Mouse Model for Chronic HAT Infection. Compound **24** was tested for *in vivo* efficacy against chronic HAT infection caused by *T. b. brucei* (TREU 667 strain) at the Haskins Laboratories, Pace University. In this assay, **24** was given orally once daily for 7 days as a suspension formulation (0.8% CMC, 0.1% SDS in water) to groups of 8 or 10 infected female Swiss Webster mice, starting 21 d postinfection, according to a reported method.[5] Animals remaining parasite free for more than 180 days beyond the end of the treatment period were considered cured.

References

1. Thompson AM, Blaser A, Anderson RF, Shinde SS, Franzblau SG, Ma Z, Denny WA, Palmer BD. *J Med Chem*. 2009; 52: 637-645.
2. Kim P, Kang S, Boshoff HI, Jiricek J, Collins M, Singh R, Manjunatha UH, Niyomrattanakit P, Zhang L, Goodwin M, Dick T, Keller TH, Dowd CS, Barry CE. *J Med Chem*. 2009; 52: 1329-1344.
3. Hayashi M, Matsuura Y, Watanabe Y. *Tetrahedron Lett.* 2004; 45: 1409-1411.
4. Kmentova I, Sutherland HS, Palmer BD, Blaser A, Franzblau SG, Wan B, Wang Y, Ma Z, Denny WA, Thompson AM. *J Med Chem.* 2010; 53: 8421-8439.
5. Nare B, Wring S, Bacchi C, Beaudet B, Bowling T, Brun R, Chen D, Ding C, Freund Y, Gaukel E, Hussain A, Jarnagin K, Jenks M, Kaiser M, Mercer L, Mejia E, Noe A, Orr M, Parham R, Plattner J, Randolph R, Rattendi D, Rewerts C, Sligar J, Yarlett N, Don R, Jacobs R. *Antimicrob Agents Chemother* 2010; 54: 4379-4388.
6. Kaiser M, Maes L, Tadoori LP, Spangenberg T, Ioset J-R. *J Biomol Screening* 2015; 20: 634-645.
7. Thompson AM, O'Connor PD, Blaser A, Yardley V, Maes L, Gupta S, Launay D, Martin D, Franzblau SG, Wan B, Wang Y, Ma Z, Denny WA. *J Med Chem.* 2016; 59: 2530-2550.

Table S2. Combustion analysis data for the compounds of Table 6 and intermediates

**No.**	**Formula**	**Calculated**			**Found**		
		**C**	**H**	**N**	**C**	**H**	**N**
**24**	$C_{14}H_{12}F_3N_3O_5S$	42.97	3.09	10.74	43.25	3.05	10.68
**26**	$C_{14}H_{12}F_3N_3O_5S$	42.97	3.09	10.74	43.16	3.00	10.70
**28**	$C_{13}H_{10}F_3N_3O_4S$	43.22	2.79	11.63	43.08	2.71	11.60
**29**	$C_{12}H_9F_3N_4O_3S$	41.62	2.62	16.18	41.90	2.51	16.11
**32**	$C_{19}H_{15}F_3N_4O_4S$	50.44	3.34	12.38	50.46	3.30	12.20
**33**	$C_{13}H_{10}F_3N_3O_5S$	41.38	2.67	11.14	41.64	2.61	11.14
**34**	$C_{12}H_9F_3N_4O_4S$	39.78	2.50	15.47	39.88	2.37	15.44
**35**	$C_{19}H_{15}F_3N_4O_5S$	48.72	3.23	11.96	48.76	3.07	11.70
**38**	$C_{14}H_{12}F_3N_3O_5S$	42.97	3.09	10.74	43.18	3.00	10.60
**40**	$C_{13}H_{10}F_3N_3O_6S$	39.70	2.56	10.68	39.98	2.61	10.61
**41**	$C_{12}H_9F_3N_4O_5S$	38.10	2.40	14.81	38.21	2.28	14.81
**54**	$C_{12}H_{11}BrN_4O_3S$	38.83	2.99	15.09	38.88	2.84	14.96
